# A hypomorphic *PIGA* gene mutation causes severe defects in neuron development and susceptibility to complement-mediated toxicity in a human iPSC model

**DOI:** 10.1371/journal.pone.0174074

**Published:** 2017-04-25

**Authors:** Xuan Yuan, Zhe Li, Andrea C. Baines, Eleni Gavriilaki, Zhaohui Ye, Zhexing Wen, Evan M. Braunstein, Leslie G. Biesecker, Linzhao Cheng, Xinzhong Dong, Robert A. Brodsky

**Affiliations:** 1 Division of Hematology, Department of Medicine, Johns Hopkins University School of Medicine, Baltimore, MD, United States of America; 2 Department of Neuroscience, Johns Hopkins University, School of Medicine, Baltimore, MD, United States of America; 3 Stem Cell Program in the Institute for Cell Engineering, Johns Hopkins University School of Medicine, Baltimore, MD, United States of America; 4 Genetic Disease Research Branch, National Human Genome Research Institute, National Institutes of Health, Bethesda, MD, United States of America; Newcastle University, UNITED KINGDOM

## Abstract

Mutations in genes involved in glycosylphosphatidylinositol (GPI) anchor biosynthesis underlie a group of congenital syndromes characterized by severe neurodevelopmental defects. GPI anchored proteins have diverse roles in cell adhesion, signaling, metabolism and complement regulation. Over 30 enzymes are required for GPI anchor biosynthesis and PIGA is involved in the first step of this process. A hypomorphic mutation in the X-linked *PIGA* gene (c.1234C>T) causes multiple congenital anomalies hypotonia seizure syndrome 2 (MCAHS2), indicating that even partial reduction of GPI anchored proteins dramatically impairs central nervous system development, but the mechanism is unclear. Here, we established a human induced pluripotent stem cell (hiPSC) model containing the *PIGA*c.1234C>T mutation to study the effects of a hypomorphic allele of *PIGA* on neuronal development. Neuronal differentiation from neural progenitor cells generated by EB formation in *PIGA*c.1234C>T is significantly impaired with decreased proliferation, aberrant synapse formation and abnormal membrane depolarization. The results provide direct evidence for a critical role of GPI anchor proteins in early neurodevelopment. Furthermore, neural progenitors derived from *PIGA*c.1234C>T hiPSCs demonstrate increased susceptibility to complement-mediated cytotoxicity, suggesting that defective complement regulation may contribute to neurodevelopmental disorders.

## Introduction

In 2006, the first germline mutation in a gene (*PIGM*) involved in GPI anchor biosynthesis was described in a child with portal and hepatic vein thrombosis and absence seizures [1]. Since then, dozens of pedigrees describing mutations in genes involved in GPI anchor biosynthesis, including the X-linked *PIGA* gene, have been described [2–9]. While the phenotypes vary, these affected individuals share a number of overlapping features. All mutations are hypomorphs, consistent with the model that complete absence of GPI anchor proteins is embryonic lethal. Overlapping clinical manifestations include early onset infantile spasms, profound developmental delay and intellectual disability, dysmorphic facial features, and multiple central nervous system abnormalities, such as thin corpus callosum, delayed myelination, and hypotonia. Severely affected patients die within the first year of life; others may survive into adulthood, but have significant intellectual disability and seizures.

Phosphatidylinositol glycan class A protein (PIGA) is one of over 30 enzymes involved in the biosynthesis of glycosylphosphatidylinositol (GPI), a glycolipid moiety that anchors more than 100 different proteins to the cell surface [10, 11]. PIGA is one of seven enzymes essential for the first step in GPI anchor biosynthesis [12]. GPI anchored proteins serve critical functions as adhesion molecules, receptors, complement regulators, enzymes and co-receptors in signal transduction pathways. The *PIGA* gene is located on chromosome Xp22.2, spans 162 kb and encodes for a widely expressed 484 amino acid protein. The remaining genes involved in GPI anchor biosynthesis are located on autosomes. Until the last decade, only somatic *PIGA* mutations had been reported in patients with paroxysmal nocturnal hemoglobinuria (PNH) [13, 14]; germline mutations had not been reported in *PIGA* or any other of the genes involved in GPI anchor biosynthesis and were suspected to result in embryonic lethality [15, 16].

PNH is a rare hematologic condition that leads to a severe complement-mediated hemolytic anemia [14, 17]. The disease develops when a hematopoietic stem cell acquires a *PIGA* mutation that leads to severe GPI anchor protein deficiency. Following clonal expansion of the *PIGA* mutant stem cell, PNH patients develop signs and symptoms that correlate with the percentage of GPI anchor deficient blood cells [18]. Hemolysis in PNH is caused by a severe deficiency of two GPI anchored complement regulatory proteins, CD55 and CD59, and the hemolytic anemia can be abrogated by a humanized monoclonal antibody to C5 that blocks terminal complement [19, 20]. Thrombosis is the leading cause of death in PNH and also correlates with the size of the PNH clone.

Germline *PIGA* null mutations are embryonic lethal due to an early block in embryogenesis, before the development of mesoderm and endoderm, that is due to loss of GPI anchored co-receptors involved in BMP4 signaling [16, 21]. In 2012, we described the first pedigree of a family with multiple congenital anomalies hypotonia seizure syndrome 2 (MIM316818, MCAHS2) due to a hypomorphic germline *PIGA* mutation (c.1234C>T) [4]. Neither patient had hemolytic anemia or clinical hemoglobinuria. The findings indicated that even subtle GPI anchor protein deficiency results in severe defects in neuronal development. Since there are limited numbers of GPI anchored proteins involved in neuron development, these rare germline mutations may offer insight into the role that specific GPI anchored proteins play in inherited and acquired neurodevelopmental and neurodegenerative diseases. Since our original report, a number other patients with inherited GPI anchor deficiency and heterogeneous neurodevelopmental congenital anomaly disorders due to hypomorphic *PIGA* mutations have been described [5, 6, 22–26].

Recently, we established a human induced pluripotent stem cell (hiPSC) model of PIGA loss of function using genomic editing to abolish function of the *PIGA* gene [16]. Differentiation of these *PIGA*null hiPSCs resulted in small embryoid bodies that did not produce blood-like cells; however, using a doxycycline inducible promoter to regulate *PIGA* expression we were able to establish GPI anchor deficient blood cells by expressing the *PIGA* gene product early in the differentiation protocol. These data, in conjunction with clinical phenotype of inherited GPI anchor deficiency syndromes, suggest that mutations that lead to reduced GPI anchor protein expression have little to no impact on hematopoiesis. However, they can produce severe defects in neuronal development and predispose to intellectual disability and seizures. In order to study the effects of partial deficiency of PIGA during neuron development, we established hiPSCs containing the hypomorphic *PIGA*c.1234C>T mutation previously described [4]. These cells have reduced expression of cell surface GPI anchored proteins and normal hematopoietic differentiation; however, they have impaired neuron differentiation, characterized by abnormal proliferation, synapse deficits, and increased susceptibility to the alternative pathway of complement.

## Materials and methods

### Cell culture

Human iPSCs were grown on irradiated mouse embryonic fibroblasts (MEF) in human embryonic stem cell (hESC) medium supplemented with 5ng/mL FGF-basic (R&D System) as we previously described. Medium was changed daily and cells were passaged every 4–7 days using collagenase IV (Life Technologies). Human TF1 cells (CRL-2003, ATCC) were cultured in RPMI 1640 medium supplemented with 10% (v/v) FBS, 1% (v/v) L-Glutamine (200mM), 1% (v/v) Penicillin-Streptomycin (Life Technologies) and 2ng/mL GM-CSF (R&D System).

### Generation of the PIGAc.1234C>T mutation in hiPSC lines

An hiPSC line derived from a healthy male was used to generate hiPSC lines with mutations in the *PIGA* gene. A complete knock out of *PIGA* was generated in hiPSCs using zinc finger nuclease (ZFN) technology as described[16]. *PIGA* gene deficiency was confirmed by lack of CD59 expression. The nonsense point mutation *PIGA*c.1234C>T was introduced into the *PIGA*null hiPSC line using the piggyBac (PB) Transposon System [4, 5]. This system was also used to introduce a wildtype *PIGA* cDNA (*PIGA*wt) and a truncated *PIGA* cDNA (*PIGA*tr411) into the PB backbone vectors for generation of hiPSC lines. *PIGA*tr411 was used as a negative vector control. All vectors were verified by DNA sequencing. *PIGA*null hiPSCs were transfected at 80% confluency with PB-*PIGA*wt, PB-*PIGA*c.1234C>T or PB-*PIGA*tr411 along with PB-Transposase using the 4D Nucleofector system (Lonza). Seventy-two hours post-transfection, CD59 expression was confirmed by flow cytometry (A15705, Molecular Probes) compared to *PIGA*tr411 as background. The remainder of transfected cells were transferred to a new coated plate and cultured in hESC medium containing 0.5–1.0 μg puromycin for selection. After 2 weeks of antibiotic selection, transfected hiPSCs were harvested and stained with anti-CD59 antibody. CD59 positive cells from *PIGA*wt and *PIGA*c.1234C>T hiPSCs were isolated using fluorescence-activated cell sorting and expanded in culture for further study. Using the same method as above, we also introduced PB-*PIGA*wt, *PIGA*c.1234C>T, and *PIGA*Tr411 into TF1*PIGA*null cells.

### Mesoderm induction and hematopoietic differentiation

Embryoid bodies (EBs) were generated from the three hiPSC lines via forced aggregation as previously described [16]. The hiPSC lines were enzymatically treated and plated in 96-well plates at a density of 3,000–5,000 cells per well in serum free medium (SFM) supplemented with 10 ng/mL FGF-basic, 10 ng/mL BMP-4, 10 ng/mL VEGF, and 50 ng/mL SCF (R&D System). The cells were aggregated by centrifugation at 3,000rpm for 5 minutes and placed in a humidified incubator at 37°C with 5% CO_2_. The medium was replaced with fresh SFM containing cytokines every 3 days. Ten days after mesoderm induction, blood-like cells (BLCs) surrounding the EBs were observed. Fourteen days after mesoderm induction, single cells from the three hiPSC lines were collected by filtration through a 70 μm filter. Cell phenotype was analyzed by flow cytometry using CD34 and CD45 as markers of hematopoietic stem cells and progenitor cells. Hematopoietic cells derived from the hiPSC lines were further cultured in suspension in SFM supplemented with 10 ng/mL Flt3, 10 ng/mL IL-3, 10 ng/mL TPO, 50 mg/mL, GM-CSF, and 50 ng/mL SCF plus 3 units/mL EPO (R & D System). After 5 days of hematopoietic differentiation, expression of CD34 (555824, BD Biosciences), CD45 (555483, BD Biosciences), CD15 (11-0159-42, eBioscience), CD33 (555450, BD Biosciences), and CD235a (559943, BD Biosciences) was analyzed by flow cytometry.

### Hematopoietic colony-forming unit (CFU) assay

Fourteen days after mesoderm induction, single cells differentiated from *PIGA*wt and *PIGA*c.1234C>T hiPSC lines were collected, counted and plated in 3 mL MethoCult^TM^ H4434 classic medium (Stem Cell Technologies) at a density of 50,000 cells per 36 mm ultra-low dish in duplicate, supplemented with 3 units/mL EPO. The dishes were kept in a humidified incubator at 37°C with 5% CO_2_ for 10 to 14 days. The CFUs were counted and characterized under an inverted microscope.

### NSC induction and neuronal differentiation

Prior to EB formation, the three hiPSC lines were cultured in a 6-well plate coated with Matrigel (BD Biosciences) in NutriStem^TM^ XF/FF medium (Stemgent). Day 0–4: When 80% confluent, hiPSCs were disassociated with Accutase (Sigma-Aldrich), counted and seeded in U-shaped 96-well plates at a density of 4,000 cells per well in 50μl of hESC medium containing 20 μM ROCK inhibitor (Y-27632, Stemgent). The cells were aggregated by centrifuging at 1500 rpm for 5 minutes, then incubated at 37 ^0^C overnight. The following day, we added 50% (50μl) of hNIM into each well (100μl final volume per well). The cells were cultured for 4 days for ectoderm induction, with 50% fresh hNIM changed daily. hNIM consists of DMEM/F12 with N-2 supplement (Life Technologies), NEAA 2 mg/mL (Sigma-Aldrich), heparin, 10 μM SB431542 and 100 ng human Noggin (R&D System). Day 4–7: 4 days after ectoderm induction, homogenous EBs derived from the three hiPSC lines (hiPSC-EBs) were pooled into a 100 mm non-treated dish and cultured in suspension in human neural progenitor medium (hNPM) with 50% fresh hNPM changed daily [27]. hNPM consists of neuronal basal medium with N-2 supplement (Life Technologies) and 100ng/mL human noggin (R&D System). Day 7–11: On day 7, floating hiPSC-EBs were re-plated in 6-well plates coated with Poly-L-Lysine (PLL) and Laminin (LM) at high density and cultured in hNPM with fresh medium replaced every other day. By day 11, neural tube-like structures and rosettes appeared in the center of hiPSC-EBs. The EB-derived rosettes were cultured in hNPM changed every other day for 4 additional days. The number of EBs containing neural rosettes (EB-derived rosettes) were counted and the percentage of neural induction was calculated using the formula: % Neural induction = # of EBS with ≥ 50% neural rosettes / total of EBs x 100 (Formula from Stemcell technologies technical manual). Day 12–19: On day 12, EB-derived rosettes were incubated with STEMdiff^TM^ neural rosette selection reagent (Stemcell Technologies) at 37^°^C for 1 hour and manually dislodged to separate them from non-neural ectoderm-like neural crest cells. The EB-derived rosettes were collected, centrifuged at 800 rpm for 3 minutes and resuspended in 2 mL hNPM. Human neural precursor cells (hNPCs) from the EB-derived rosettes were re-plated in a 6-well plate coated with PLL/LM and cultured in hNPM for 5–7 days (with medium changed daily) until 80–90% confluent, then expanded and frozen, or subjected to further neuronal differentiation. The proliferation of hNPCs derived from *PIGA*wt, *PIGA*c.1234C>T, and *PIGA*null hiPSC lines, was measured using the Click-iT® EdU Alexa Fluor® 488 Imaging Kit (Thermo Fisher Scientific). Neuronal stem cells and progenitor cells were characterized by immunofluorescence staining with antibodies against SOX1 (AF3369, R&D System), Nestin (MAB 1259, R&D System) and PAX6 (Ab5790, Abcam). Day 19: 80–90% confluent hiPSC-derived hNPCs were dissociated into single cells with Accutase (Sigma) at 37 ^0^C for 5 minutes, counted, split 1:4 and transferred to a new 6-well plate coated with PLL/LM for neuronal differentiation. For neuronal differentiation, German glass coverslips (NeuVitro) were coated with PLL/LM (Sigma) in 24-well plates and incubated at 4 ^0^C overnight, washed three times with sterile water and air dried. Mouse astrocytes were seeded as a feeder layer. 100μL of inactive mouse astrocyte suspension P2 (M1800, ScienCell) was applied to the PLL/LM-coated coverslips and incubated at 37 ^0^C for 2 hours to promote attachment before addition of mouse astrocyte medium (ScienCell). Human NPCs derived from the three hiPSC lines were plated onto PLL/LM-coated coverslips or mouse astrocyte feeder coverslips, at low density in hNPM in a 37 ^0^C incubator. The next day, hNPM was replaced with Neuronal differentiation medium (NDM) supplemented with 10 ng/mL BNDF and GNDF (R&D System) and 1 mM cyclic AMP (Sigma). NDM consists of Neurobasal medium (Life Technologies) with 1x B27 supplement (Life Technologies) and 1:100 GlutaMax (Life Technologies). The cells were cultured in NDM for 4–6 weeks and fresh NDM was added twice a week. hNPCs grown on mouse astrocyte feeders were cultured in NDM with fresh NDM added twice a week. After 2 to 4 weeks of neuronal differentiation, the neurons from *PIGA*wt, *PIGA*c.1234C>T and *PIGA*null NPCs were used for electrophysiology studies.

### Electrophysiological recording

Whole-cell patch-clamp recordings were performed on 2-week-old hiPSC neurons. Parallel cultures were used for recordings of all three hiPSC NPC lines. The recording chamber was perfused with fresh HEPES-buffered saline composed of 140 mM NaCl, 5 mM KCl, 2 mM CaCl_2_, 2 mM MgCl_2_, 10 mM HEPES, and 10 mM glucose, pH adjusted to 7.4 with NaOH, osmolarity adjusted to 300 mOsm with sucrose [28]. The recording micropipettes (tip resistance 3–6 MΩ) were pulled (Model pp-830, Narishige) from borosilicate glass (WPI Inc.). Internal solution containing 135 mM KCl, 1.1 mM CaCl_2_, 10 mM HEPES, 2 mM EGTA, 3 mM Mg-ATP, 0.5 mM Na_4_GTP, pH adjusted to 7.3 with KOH, osmolarity adjusted to 290 mOsm with sucrose. Recordings were made using an Axopatch 200B amplifier (Axon Instruments). Signals were sampled at 10 kHz and filtered at 2 kHz. With voltage-clamp whole-cell configuration (holding at -60 mV), currents were measured with an Axon 700B amplifier and the pCLAMP 9.2 software package (Molecular Devices) in order to characterize Na^+^/K^+^ current. With current-clamp whole cell configuration, step currents (100 to 900 pA, 100 pA apart) were injected to elicit action potentials. Each injected current lasted 200 ms (or 500 ms) and separated by an interval of 3 s. All recordings were performed at room temperature.

### Flow cytometry

*PIGA*wt, *PIGA*c.1234C>T and *PIGA*tr411 hiPSCs were stained with an Allophycocyanin (APC)-conjugated anti-human CD59 antibody (NB500-400APC, clone MEM-43/5, Novus Biologicals). CD59 is a GPI anchored protein and was used as an indicator of functional GPI anchor production. Cells were incubated with antibody on ice for 30 minutes and analyzed by flow cytometry (BD Biosciences). Hematopoietic cells were stained with APC-conjugated mouse anti-human CD34 (555824, BD Biosciences), PE-conjugated mouse anti-human CD45 (555483, BD Biosciences), FITC-conjugated mouse anti-human CD15 (11-0159-42, eBioscience), PE-conjugated mouse anti-human CD33 (555450, BD Biosciences), FITC-conjugated mouse anti-human CD71 (555536, BD Biosciences), and FITC-conjugated mouse anti-human CD235a (559943, BD Biosciences) by incubating on ice in the dark for 30 minutes and analyzed by flow cytometry (BD LSRII flow cytometry system, BD Biosciences). Samples were gated based on physical parameters (forward and side scatter). Gating for FITC-conjugated and APC-conjugated antibodies was based on the isotypic control antibodies and was confirmed by reference to negative controls. FACS data was analyzed using FlowJo Software (Treestar Inc.).

### Immunocytochemistry

Cells were fixed with 4% paraformaldehyde for 15 minutes and permeabilized and blocked with 10% donkey serum with 0.3% Triton X-100 (Sigma) in PBS for 30 minutes. Samples were incubated with primary antibodies at 4 ^0^C overnight, then incubated with appropriate secondary antibodies for 1 hour and stained with Hoechst 33342. Images were taken using a Zeiss confocal microscope (Zeiss LSM510 META) and analyzed with Zen imaging software (Zen 2012 blue edition) and ImageJ (NIH). Cell proliferation of hNPCs during neural induction was assayed using the EdU Alexa Imaging Kit (Thermo Fisher). Fluorescent cells were counted and the percentage of positive cells was calculated relative to the total number cells in the field. Following Edu staining, the hNSCs and hNPCs derived from hiPSCs were characterized by immunofluorescence for SOX1 (AF3369, R&D System) and PAX6 (Ab5790, Abcam). Cells positive for SOX1 or PAX6 were counted manually and the percentage of positive cells was calculated and normalized to the total number of cells per given image field. At least 20 image fields from three slides per cell line were counted. Four-week-old neurons were characterized using mouse anti-MAP2 (1:10,000; M2320, Sigma), rabbit anti-GABA (1:500; A2052, Sigma), mouse anti-synapsin (1:500; 106001, Synaptic System), VGLuT1 (1:500; 135303, Synaptic System), and VGAT (1:500; 131003, Synaptic System). Images were acquired with the same settings for all three cell lines. For analysis of synaptic density, total VGluT1+ in a given image was counted using ImageJ Analyze Particles and VGluT1+ density was determined by using identical settings across multiple images. The numbers of VGAT+ and VGluT1+ were also manually counted. The density of VGAT+ was determined per 100 μM total dendritic length.

### Western blot

The three hiPSC lines were incubated with or without BMP4 (50 ng/mL) for 4 hours. Protein was extracted as previously described [16] and concentration was determined by BCA assay (Pierce). Fifteen μg of protein was separated by SDS- PAGE (Invitrogen) and transferred to Hybond-P PVDF membrane (Amersham). The membrane was blocked with 5% non-fat dried milk in TBST for 30 minutes, incubated with diluted PhosphoSmad1/Smad5/Smad8 antibody (9511S, Cell Signaling Technology) in 5%(w/v) BSA, 1xTBST at 4°C with gentle shaking overnight, then incubated with HRP-conjugated donkey anti-rabbit IgG (1:2,000 in TBST, Cell Signaling Technology) at ambient temperature for 1 hour. Reactive protein bands were visualized using ECL plus Western blotting detection reagents (GE Healthcare) and band intensities were calculated using the Bio Rad Chemi Doc XRS imaging system. Beta-actin (13E5) Rabbit mAB (4970, Cell Signaling Technology) was used for a loading control.

### Complement-mediated cytotoxicity assay

The complement-mediated cytotoxicity assay was performed based on a cell viability assay that has been described [27]. *PIGA*wt, *PIGA*c.1234C>T and *PIGA*null hNPCs were incubated with normal human serum (Sigma-Aldrich), normal human serum containing cobra venom (0.5 μg/mL, Complement Technology, Tyler, TX) to activate the alternative pathway of complement, and human serum from a patient with atypical hemolytic uremic syndrome (aHUS) where there is constitutive overactivity of the alternative pathway of complement. Sera were diluted (20%) in gelatin veronal buffer (GVB) (Sigma-Aldrich) and incubated with TF1*PIGA*null cells for 30 minutes at 37°C. The cells were then washed with PBS and incubated with WST-1 cell proliferation reagent (Roche) for 2 hours at 37°C. Absorbance was measured in an iMark Microplate Absorbance Reader (Bio-Rad, Hercules, CA) at 490 nm with a reference wavelength at 595 nm. Heat-inactivated serum was used as a negative control. The percentage of non-viable cells was calculated using the formula: 100 - (sample absorbance x 100 / heat inactivated sample’s absorbance).

## Statistics

Statistics were performed using GraphPad Prism 6 (GraphPad Software). A one-tailed, unpaired Student’s *t* test, Mann-Whitney *U* test, or one way ANOVA and multiple comparisons was used as appropriate. An F test was performed in Prism to determine whether variances were similar among groups. A *P* value of less than 0.05 was considered statistically significant.

## Results

### PIGAc.1234C>T is a read-through mutation

**Fig 1:**
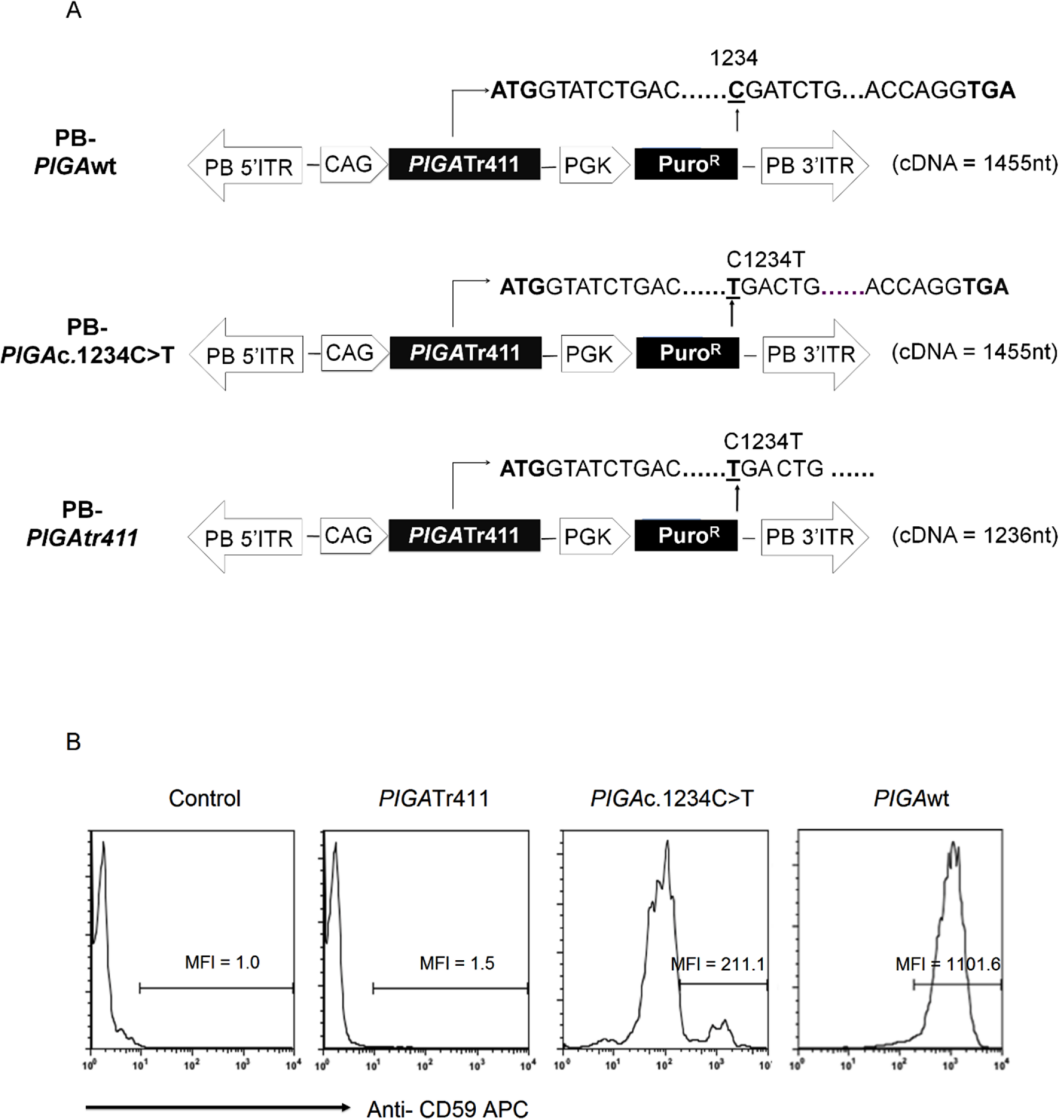
Generation of *PIGAc*.1234C>T mutation using the PiggyBac transposon system. (A). Map of PiggyBac (PB) constructs for *PIGA*wt (top), *PIGA*c.1234C>T (middle) and *PIGA*tr411 (bottom). c.1234C>T (p.Arg412*) is a nonsense point mutation in the 6^th^ exon of the *PIGA* gene that predicts a truncated protein missing the final C-terminal 73 amino acids. *PIGA*tr411 is the truncated form of the *PIGA* cDNA, lacking the coding sequence of the C-terminal 73 amino acids. (B). Representative FACS analysis CD59 expression in TF1*PIGA*null cells transfected with PB-*PIGA*wt, PB-*PIGA*c.1234C>T or PB-*PIGA*tr411. Transfected TF1*PIGA*null cells were stained with an APC-conjugated CD59 antibody to assess *PIGA* gene expression. Non-transfected TF1*PIGA*null cells were used as a control. MFI represents mean fluorescence intensity.

Germline *PIGA* null mutations are embryonic lethal and lead to a block in mesodermal and endodermal differentiation due to decreased BMP4 signaling. The *PIGA*c.1234C>T mutation associated with inherited GPI anchor protein deficiency occurs in the last exon of *PIGA* and is predicted to result in a truncated protein missing the final C-terminal 73 amino acids; thus, we were initially surprised to find this was a hypomorphic mutation in a male patient [4]. To investigate further, we used an expression vector and stably transfected TF1 *PIGA*null cells with either wildtype *PIGA* full-length cDNA (*PIGA*wt), a full-length *PIGA* cDNA containing the c.1234C>T mutation (*PIGA*c.1234C>T), or a truncated *PIGA* cDNA (*PIGA*tr411*)* encoding only the first 411 amino acids as predicted based on the nonsense c.1234C>T mutation (Fig 1A). The reconstitution assay shows that the *PIGA*c.1234C>T cDNA rendered a reduced, but detectable, level of GPI anchor protein expression (Fig 1B). The hypomorphic state is likely due to “read-through” of the premature stop codon. In contrast, the truncated *PIGA*tr411 cDNA (lacking the final 73 amino acids) was unable to restore GPI anchor protein expression.

### The PIGAc.1234C>T mutation does not impair hematopoiesis

**Fig 2:**
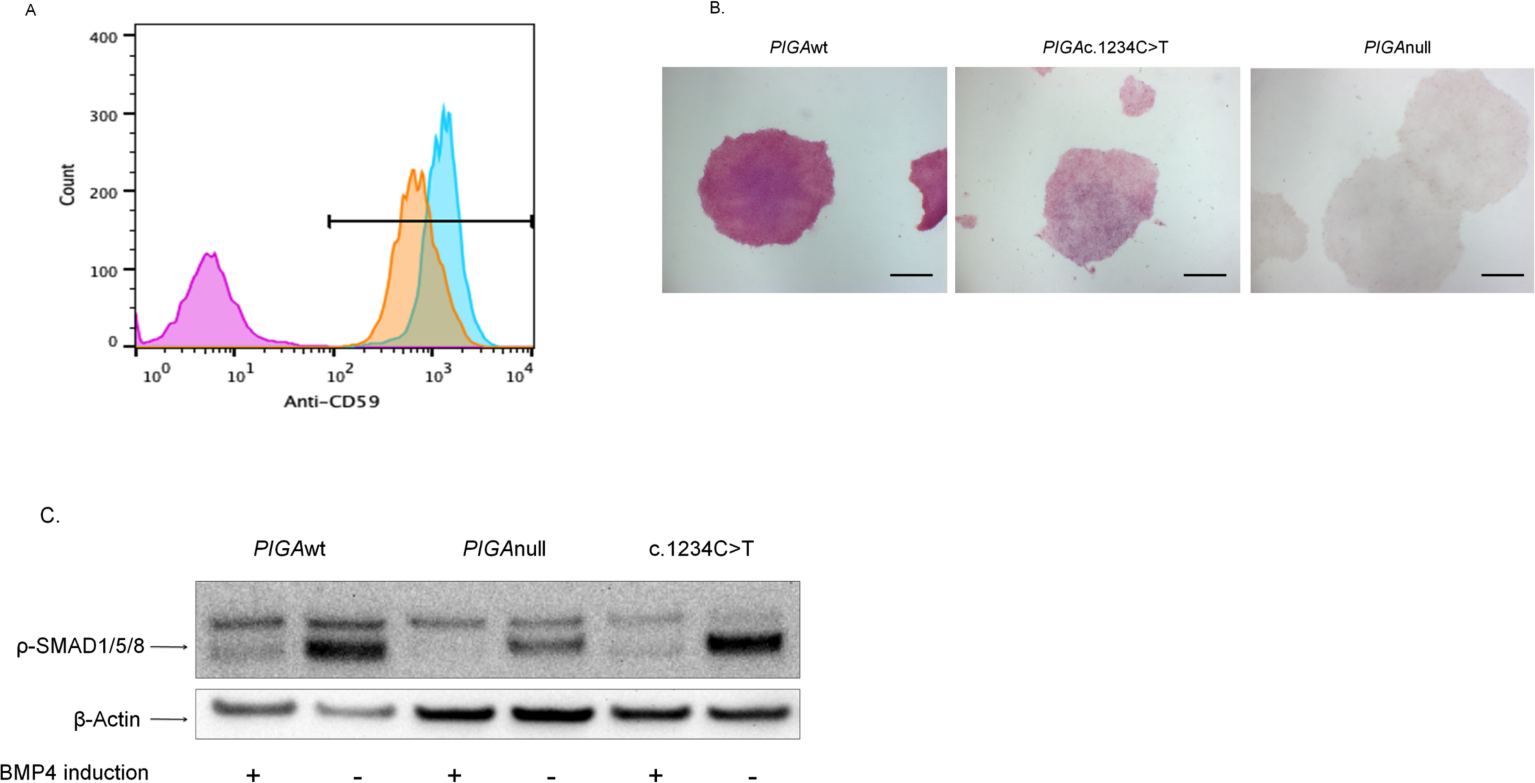
The *PIGA*c.1234C>T mutation increases *PIGA* function compared to *PIGA*null hiPSCs. (A). Representative example of FACS analysis CD59 expression in the three hiPSC lines. Overlay histogram shows that CD59 expression was significantly higher in *PIGA*c.1234 C>T hiPSCs compared to *PIGA*null hiPSCs. MFI was 445.4 in *PIGA*wt hiPSCs and 332.6 in *PIGA*c.1234C>T hiPSCs (p>0.05, NS). However, MFI in *PIGA*c.1234C>T hiPSCs was significantly higher than 17.9 in *PIGA*null hiPSCs (*p<0.05). The results indicated *PIGA* gene function was partially restored in *PIGA*c.1234C>T hiPSCs. *PIGA*null hiPSCs (purple), *PIGA*c.1234C>T hiPSCs (orange) and *PIGA*wt hiPSCs (blue). (B). Representative example of Alkaline Phosphatase (AP) activity in the three hiPSC lines was detected by an Alkaline Phosphatase Detection Kit. AP activity was examined under light microscopy (20X magnification, scale bar 50μm). AP activity was increased in *PIGAc*.*1234C>T* hiPSCs compared to *PIGA*null hiPSCs. Further confirming *PIGA*c.1234C>T hiPSCs possess partial *PIGA* gene function. (C). Representative Western blot: BMP4 induction in three hiPSC lines. *PIGA*wt, *PIGA*null and *PIGA*c.1234C>T, were treated with or without 50ng/mL BMP4 for 4 hours and levels of phosphoSmad1 (Ser463/465)/Smad5 (Ser463/465)/Smad8 (Ser426/428) were detected by immunoblotting. Intensity of Smad 1/5/8 phosphorylation was comparably increased following BMP4 induction in both of PIGA*wt* hiPSCs and *PIGA*c.1234C>T hiPSCs; decreased responsiveness to BMP4 induction was observed in *PIGA*null hiPSCs as previously described. β -actin was used as an internal control.

**Fig 3:**
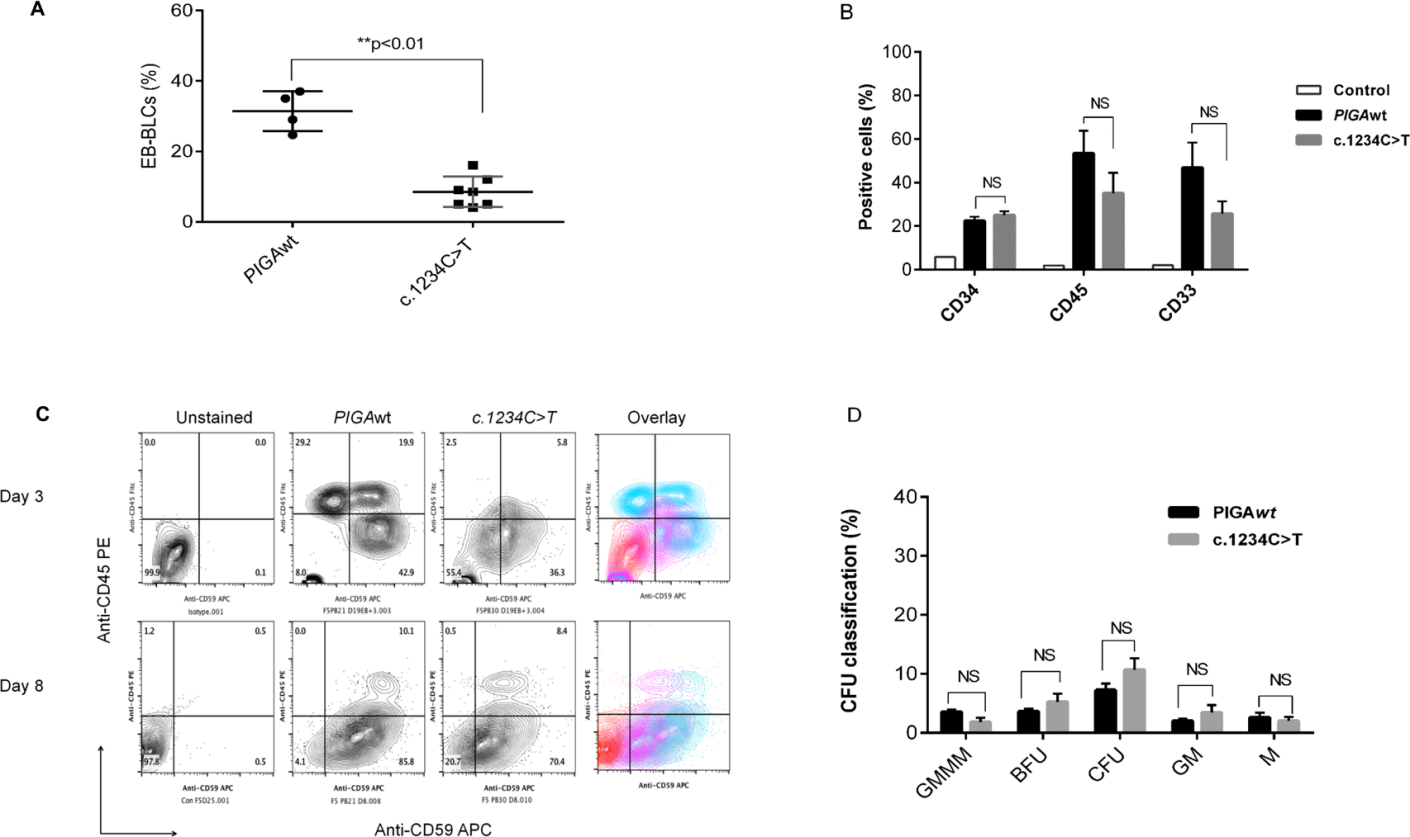
The *PIGA*c.1234C>T mutation does not impair terminal hematopoietic differentiation during mesoderm induction. (A). Quantitation of EB-derived blood-like cells (EB-BLCs). A total of 100 hiPSC-derived EBs were assessed for each hiPSC derived cell line and the percentage of EB-BLCs present was determined. The percentage of EB-BLCs derived from *PIGA*wt hiPSCs was significantly higher than EB-BLCs from *PIGA*c.1234C>T hiPSCs (**P<0.01, Mann-Whitney test). Results shown are average and standard deviation (mean ± SD) based on three independent experiments. (B). Expression of hematopoietic markers CD33, CD34 &CD45 in EB-BLCs from *PIGA*wt and *PIGA*c.1234C>T hiPSCs. The cells were stained with anti-human CD33, anti-human CD34 and anti-human CD45 for flow cytometry analysis. Antibodies and analyzed by flow cytometry. The values shown are from three independent experiments. All values represent average and standard error (mean ± SE). (C). Representative example of FACS analysis of hematopoietic phenotypes in the EB-BLCs from *PIGA*wt and *PIGA*c.1234C>T. The zebra plot shows expression of CD59 (X-axis) and CD45 (Y-axis) after three and eight days of hematopoietic differentiation. Unstained *PIGA*wt cells were used as a control. (D). Enumeration of colony forming units (CFU) from the BLCs derived from *PIGA*wt and *PIGA*c.1234C>T. There was no significant difference in CFU colony formation between *PIGA*wt and *PIGA*c.1234C>T hematopoietic cells (p>0.05, NS, one-tailed, Unpaired T test). All values represent mean ± SE. Abbreviations: CFU-Macrophage (M); CFU-Granulocyte-Macrophage (GM); committed erythroid BFU-E (BFU) and CFU-E (CFU) progenitors; multipotent progenitor cells CFU-GEMM (GEMM).

To assess the effect of the *PIGA*c.1234C>T mutation on hematopoiesis, we used a piggyBac (PB) transposon-based gene transfer system to introduce the mutation into a previously established *PIGA*null hiPSC line and differentiated the three hiPSC lines (*PIGA*wt, *PIGA*c.1234C>T, and *PIGA*null) toward mesoderm. The *PIGA*c.1234C>T hiPSCs were hypomorphic for the GPI anchored proteins CD59 (Fig 2A) and alkaline phosphatase (Fig 2B). We previously showed that *PIGA*null hiPSCs do not generate hematopoietic cells [16]. The level of phosphorylated Smads (forms 1/5/8) upon BMP4 induction was not significantly different between the *PIGA*c.1234C>T hiPSCs and the *PIGA*wt hiPSCs (Fig 2C). The hypomorphic *PIGA*c.1234C>T hiPSCs generated significantly fewer EB-derived blood-like cells (Fig 3A), but were able to generate a similar percentage of CD34+CD45+ hematopoietic cells (Fig 3B and 3C) and similar numbers of hematopoietic colony forming cells as the *PIGA*wt hiPSCs (Fig 3D). Taken together, the hypomorphic *PIGA*c.1234C>T mutation associated with germline GPI anchor deficiency is permissive for hematopoiesis, and this appears to explain why children born with germline mutations that lead to partial GPI anchor protein expression have no demonstrable hematopoietic defects.

**Fig 4:**
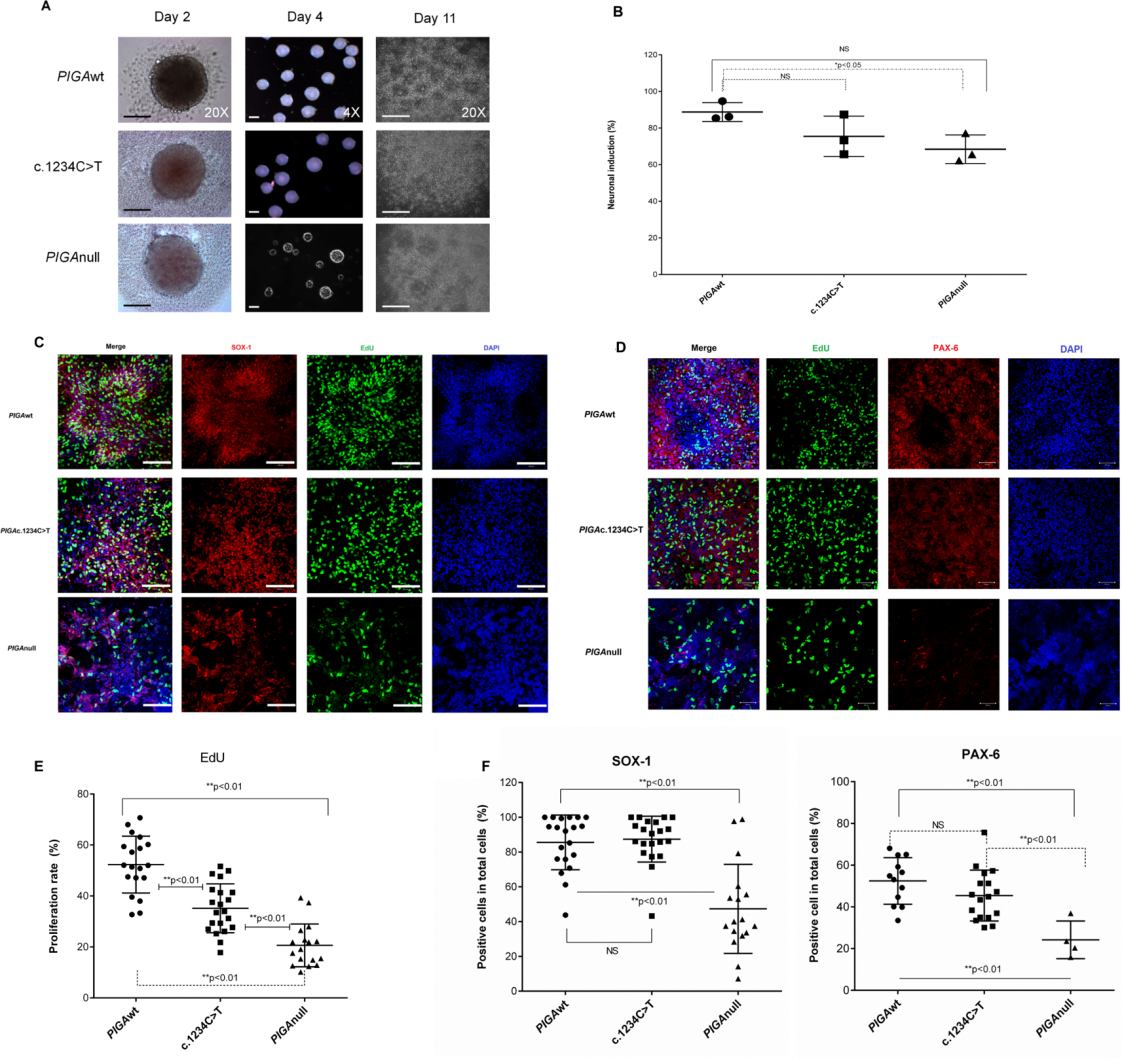
GPI anchored proteins are required for neural differentiation. (A). Representative example of images of hiPSC-derived EBs and EB-derived rosettes during neural differentiation. Neural induction and rosette formation upon neural induction was assessed in three cell lines using a serum-free EB generation method. On day 2 (left) of hiPSC-derived EBs from *PIGA*wt, *PIGA*c.1234C>T, and *PIGA*null after forced aggregation (20X magnification, scale bar is 50μm). On day 4 (middle), single homogeneous hiPSC-EBs collected were pooled in a 10 cm plate (4X magnification, scale bar is 100μm). On day 11 (right), neuroepithelial cells appeared and neural tube-like rosettes formed (EB-derived rosettes) and scale bar is 50μm. (B). Neural induction rates from EB-derived rosettes. The percentage of EB derived rosettes was 88.8% ± 4.6, 75.5% ± 9.8 and 68.4% ± 6.9 for *PIGA*wt, *PIGA*c.1234C>T, and *PIGA*null, respectively. *PIGA*wt versus *PIGA*c.1234C>T (p>0.05, NS) and *PIGA*wt versus *PIGA*null (*p<0.05, one way ANOVA and Multiple comparisons). Neural induction from *PIGA*null hiPSCs was less than 70%. All values were mean ±SD. (C). Representative confocal images showing expression of neuron stem cell marker SOX1 (in red) combined proliferation by EdU labeling in hNPCs derived from isolated neural rosettes. Nuclei were visualized with DAPI (blue) and scale bar 100μm. (D). Representative confocal images showing expression of neuron progenitor marker PAX6 (in red) and combined proliferation by EdU (in green) in hNPCs derived from isolated neural rosettes. Nuclei were visualized with DAPI (blue) and scale bar 200μm. hNPCs from *PIGA*null cell lines showed reduced expression of SOX1 and PAX6. (E). Proliferation rate in hNPCs was assessed and plotted in all three cell lines. EdU positive cells were counted and normalized by total number of nuclei staining with DAPI (blue). Proliferation was significantly decreased in *PIGA*null and *PIGA*c.1234C>T compared to *PIGA*wt. (F)**.** Graphs depict the percentage of positive cells for SOX-1 (left) and Pax6 (right) in hNPCs derived from *PIGA*wt, *PIGA*c.1234C>T, and *PIGA*null hiPSC lines. The hNPCs derived from the *PIGA*null hiPSCs showed significantly decreased expression of SOX1 and PAX6. Similar levels of SOX1 and PAX6 were expressed in hNPCs from *PIGA*wt and *PIGAc*.1234C*>*T. All values represent mean ± SD.

**Fig 5:**
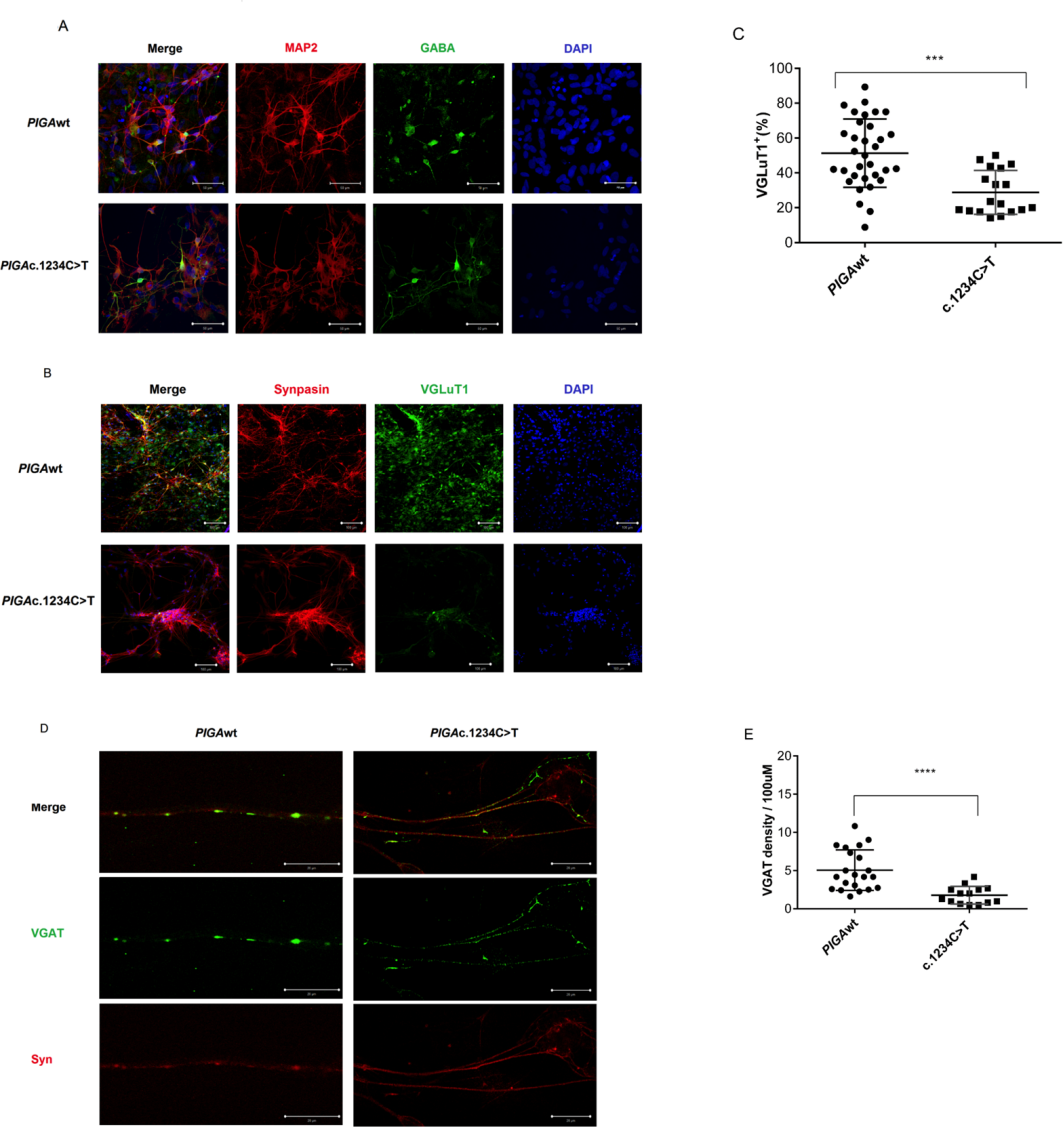
Characterization of human neural progenitor cell (hNPC) derived neurons during neuronal differentiation. (A). Representative confocal scan images of MAP2 and GABA neurons. Four weeks after neuronal differentiation, hNPC-derived neurons from *PIGA*wt and *PIGA*c.1234C>T hNPCs were stained with anti-human MAP2 (microtubule-associated protein 2, red) and anti-human GABA (γ-amino butyric acid, green); nuclei were stained with DAPI (blue). Scale bars represent 50μm. Comparison of hNPC-derived neurons from *PIGA*wt (top) and *PIGA*c.1234C>T (bottom) showed dramatically reduced gro*wt*h in the neurons from the *PIGA*c.1234C>T cell line (S2 Fig). (B). Representative confocal images of immunofluorescence staining for Synapsin (red) and VGLuT1 (vesicular glutamate transporter-1, green) in hNPC-derived neurons from *PIGA*wt and *PIGA*c.1234C>*T* cell lines. After 4 weeks of neuronal differentiation, neuron proliferation was markedly decreased in *PIGA*c.1234C>T. The density of Synapsin and VGLuT1 was substantially decreased in hNPC-derived neurons from *PIGA*c.1234C>T compared to the *PIGA*wt cell line. Scale bars represent 100 μm. (C). Quantification of VGLuT1 density in hNPC-derived neurons from *PIGA*wt and *PIGA*c.1234C>T. The number of VGLuT1 positive neurons was counted and plotted. Values represent the mean ± SD. (D). Representative confocal images showing the density of VGAT (vesicular GABA transporter, green) and Synapsin (red) in hNPC-derived neurons from *PIGA*wt and *PIGA*c.1234C>T cell lines. There was decreased density of VGAT and synapse formation in the hNPC-derived neurons from *PIGA*c.1234C>T cells compared to the control cell line (*PIGA*wt) (S3 Fig). Scale bars represent 20 μm. (E). Density of VGAT per 100 μm in *PIGA*wt and *PIGA*c.1234C>T derived neurons. Values represent the mean ± SD.

GPI anchor proteins are important for neuron development. Common features of inherited GPI anchor deficiency are severe intellectual disability, seizures, and other central nervous system abnormalities; thus, we next studied neuronal differentiation of the hiPSCs (S1 Fig). PIGAwt, PIGAc.1234C>T, and PIGAnull hiPSCs were differentiated into neural progenitor cells. By day 11 of neuronal differentiation, neural tube-like structures (rosettes) appeared in the center of hiPSC-EBs, but the PIGAnull hiPSCs formed fewer neuron rosettes (Fig 4A) and were less capable of differentiating into cells of neuronal fate (Fig 4B). We next measured cell expression of SOX1 (a marker of neural stem cells) (Fig 4C and 4F), PAX6 (a marker of neural progenitor cells) (Fig 4D and 4F), and proliferation using EdU (Fig 4E) in the neural progenitor cells derived from the three cell lines. A PIGA gene dosage effect was observed with the PIGAc.1234C>T derived neural progenitors demonstrating intermediate proliferation and the PIGAnull derived neural progenitors showing virtually no proliferation (Fig 4E). Next, we plated the detached PIGAwt and PIGAc.1234C>T derived rosettes in NDM medium and maintained them in culture for two to four weeks; PIGAnull rosettes were not capable of neuron differentiation (data not shown). After four weeks of neuron differentiation, a phenotypic distinction in synapse formation and proliferation was observed between the PIGAwt and PIGAc.1234C>T derived neurons (Fig 5A). Consistent with the proliferation data, the GABA density of neurons derived from the PIGAc.1234C>T hiPSCs was much less due to reduced maturation of the PIGAc.1234C>T derived MAP2 and GABA expressing neurons (S2 Fig). To examine synapse morphology, we co-immunostained neurons with presynaptic markers, Synapsin and VGLuT (Fig 5B), and Synapsin and VGAT (Fig 5C). Quantification of VGLuT (Fig 5D) and VGAT (Fig 5E) revealed reduced synapse formation in the PIGAc.1234C>T derived neurons compared to the PIGAwt derived neurons. Taken together, these studies suggest that GPI anchor deficient neurons have significantly reduced proliferation, reduced maturation and presynaptic defects (S3 Fig).

### GPI anchor protein deficient neurons exhibit impaired membrane depolarization

**Fig 6:**
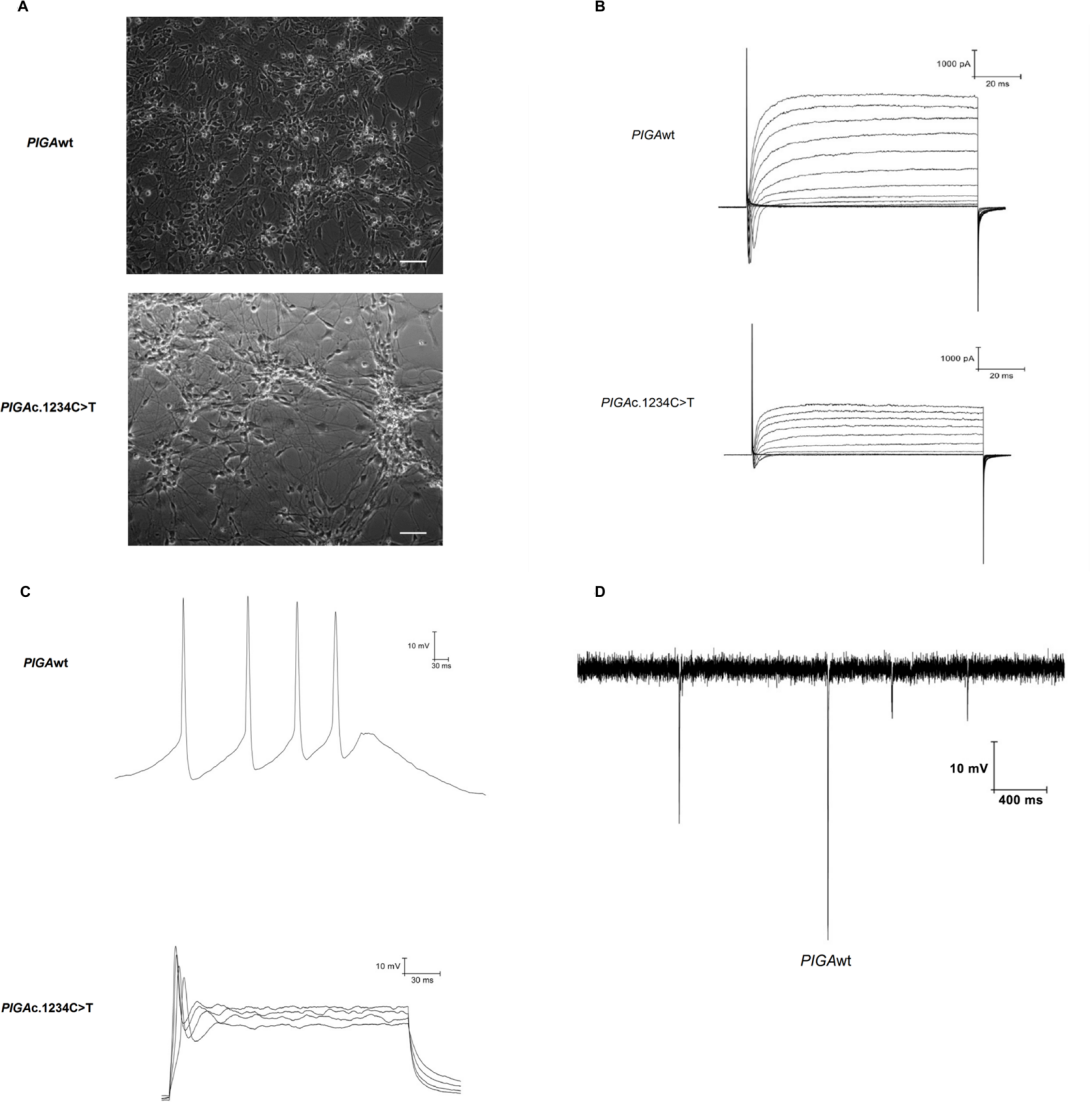
Electrophysiological characterization of neurons derived from *PIGA*c.1234C>T hNPCs revealed a severe defect in neuronal activity. Representative example of (A). Light microscopy images of hNPC derived neurons from *PIGA*wt (top), and *PIGA*c.1234C>T (bottom) hiPSC lines after 2 weeks of neuronal maturation (20X magnification, scale bar, 50μm). (B). Measurement of spontaneous neuronal activity by electrophysiology. Whole cell patch-clamp recording was performed on 2-week-old hiPSC derived neurons. *PIGAwt* neurons showed normal transit of inward sodium (Na^+^) currents and sustained outward potassium (K^+^) currents in response to membrane depolarization. Resting membrane potential was approximately -40 mV. *PIGAc*.1234C>T neurons showed normal Na^+^/K^+^ currents, though with reduced amplitude compared *PIGA*wt neurons. (C). Induction of action potential in hiPSC derived neurons by current injection. Multiple action potential peaks were observed in *PIGA*wt neurons. A single action potential peak with smaller amplitude was observed in *PIGA*c.1234C>T neurons. (D). Sample trace of excitatory spontaneous synaptic currents in *PIGA*wt neurons.

We next measured induced action potentials with whole-cell patch-clamp recordings performed on two-week-old neurons derived from the hiPSCs (Fig 6A). As shown in Fig 6B, the *PIGA*wt neurons showed normal transient inward sodium currents and sustained outward potassium currents in response to membrane depolarization. The *PIGA*c.1234C>T neurons exhibited a decrease in both currents. The *PIGA*null neurons showed almost no sodium current and markedly reduced potassium currents. The number and amplitude of action potentials was also reduced in the *PIGA*c.1234C>T neurons compared to the *PIGA*wt neurons (Fig 6B), further highlighting the synaptic defect associated with GPI anchor protein deficiency. No depolarizations were observed in the *PIGA*null line, consistent with our data above demonstrating an inability of this cell line to differentiate into mature neurons (Fig 6C). Spontaneous excitatory postsynaptic currents (EPSCs) were observed only in the *PIGA*wt neurons, indicating an impaired neuronal property of *PIGA*c.1234C>T neurons and inability of *PIGA*null hiPSCs to properly differentiate (Fig 6D).

### PIGAc.1234C>T derived neurons are more vulnerable to complement-mediated cytotoxicity

**Fig 7:**
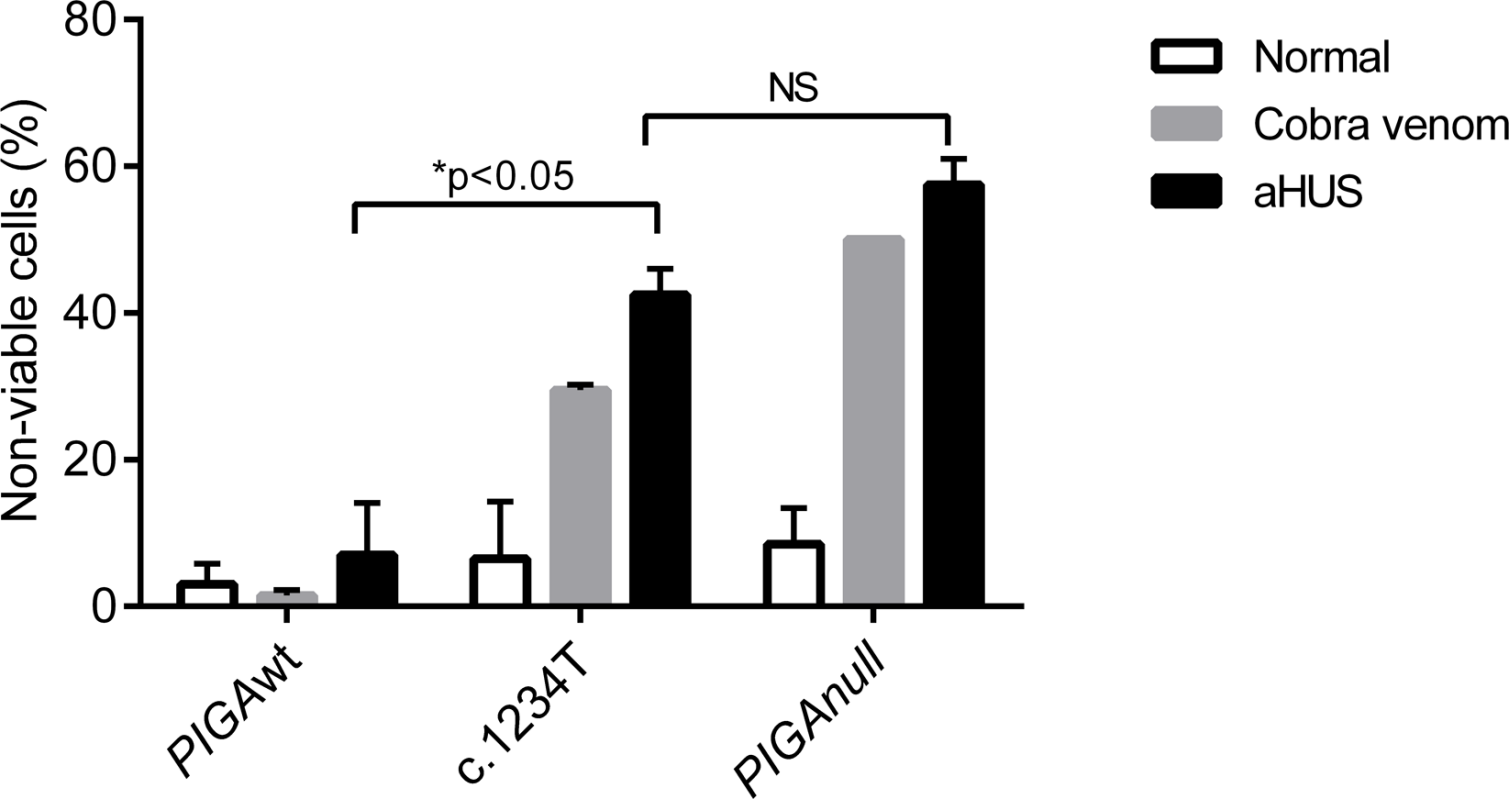
Susceptibility of hNPCs to complement-mediated cytotoxicity. The complement-mediated cytotoxicity assay was performed in *PIGA*wt, *PIGA*c.1234C>T, and *PIGA*null hNPCs. The cells were incubated with normal serum, cobra venom factor or atypical HUS serum for 30 minutes. The percentage of non-viable cells was measured using WST-1 cell proliferation reagent. *PIGA*null hNPCs were the most susceptible to complement-mediated cell killing, followed by *PIGAc*.1234C>T cells, which were more susceptible to complement-mediated killing compared to *PIGA*wt cells (p<0.05). Values were mean ± SD. NS (normal human serum, black), CVF (cobra venom factor, patterned), aHUS (serum from a patient with atypical hemolytic-uremic syndrome, white).

GPI anchored proteins CD55 and CD59 are important complement regulatory proteins, suggesting that complement activation could play a pathophysiologic role in the neurodegeneration and or seizures associated with inherited GPI anchor protein deficiency. To test the ability of GPI deficient neuronal stem cells to withstand complement mediated attack, we exposed day 17 neuronal progenitor cells derived from *PIGA*wt, *PIGA*c.1234C>T and *PIGA*null hiPSCs to 20% normal human serum, 20% normal human serum containing cobra venom (0.5 μg/mL) to activate the alternative pathway of complement, or 20% human serum from a patient with atypical hemolytic uremic syndrome (aHUS), where there is constitutive activation of the alternative pathway of complement [29, 30]. A *PIGA* gene dosage effect was again observed, with *PIGA*c.1234C>T and *PIGA*null derived neural stem cells demonstrating a marked reduction in cell viability following exposure to activated alternative pathway of complement (Fig 7).

## Discussion

We described here a model of germline GPI anchor protein deficiency using human iPSC lines. Germline mutations that lead to a subtle decrease in cell surface display of GPI anchored proteins are increasingly being recognized to cause inherited syndromes that manifest with dysmorphic faces, intellectual disability, and intractable seizures. Here, we generated a human model of germline GPI anchor protein deficiency by establishing a human iPSC line containing a hypomorphic mutation *PIGA*c.1234C>T that mimics the human disease. *PIGA*c.1234C>T hiPSCs had no substantive hematopoietic defects upon hematopoietic differentiation; however, upon neuronal differentiation, they had significant defects in proliferation and synapse formation. Moreover, GPI anchor deficient neuronal stem cells derived from the *PIGA*c.1234C>T human hiPSCs were more susceptible to complement-mediated cytotoxicity in an *in vitro* assay exposing them to activation of the alternative pathway of complement, presumably due to deficiency of GPI anchored complement regulatory proteins.

Acquired *PIGA* mutations arising from a multipotent hematopoietic stem cell cause PNH and lead to chronic complement-mediated hemolysis due to marked deficiency or absence of GPI anchored complement regulatory proteins CD55 and CD59 [13, 31]. Compound heterozygous mutations (one inherited and one acquired) in *PIGT* and germline mutations involving CD59 also lead to a PNH-like phenotype with severe anemia from marked intravascular hemolysis [32]. Moreover, complete knock-out of *Piga* in mice is embryonically lethal [33] and knockout of *PIGA* in human iPSCs does not allow for hematopoietic differentiation due to perturbed signaling through BMP4[16]. Interestingly, none of the reported patients with germline mutations in *PIGA* or any of the other genes involved in GPI anchor biosynthesis have exhibited any hematopoietic defect or hemolysis. Similarly, the *PIGA*c.1234C>T hiPSCs demonstrated no defect in hematopoiesis. The lack of hemolysis in these patients is likely due to 1) the very subtle defect (~ a quarter of a log decrease) in cell surface GPI anchored proteins, and 2) the fact that red blood cells from most patients with germline GPI mutations have normal expression of CD55 and CD59; the defect is most conspicuous on granulocytes [1, 4, 34].

Despite the subtle decrease in GPI anchor proteins observed in the *PIGA*c.1234C>T hiPSCs and primary cells of patients with germline hypomorphic mutations in *PIGA* and other GPI-related genes, the neurologic phenotype is severe. Neurons derived from the *PIGA*c.1234C>T iPSCs displayed a defect in cell proliferation. In addition, neurons derived from *PIGA*c.1234C>T iPSCs displayed a decrease in presynaptic markers Synapsin, VGAT, and VGLuT, possibly explaining the CNS imaging abnormalities, seizures, and severe intellectual disability associated with these disorders. Patch-clamp studies showed that the *PIGA*c.1234C>T derived neurons had a reduction in the ability to fire action potentials, a reduction in amplitude of sodium and potassium currents and a reduction in spontaneous EPSCs, further demonstrating the neurodevelopmental defects and synaptic dysfunction associated with reduced GPI anchor protein expression.

There are more than a dozen known GPI anchored proteins that are expressed during neurogenesis that could be responsible for the neurodevelopmental defects associated with GPI anchor protein deficiency. Members of the nerve growth factor (NGF) and glial cell line-derived neurotrophic factor (GDNF) families are crucial for the development and maintenance of the central and peripheral nervous system [35]. NGF and GDNF are added to our neuronal stem cells in culture on day 19 to promote proliferation and differentiation. Receptors (GFRα1, GFRα2, GFRα3, and GFRα4) for GDNF-family ligands are GPI anchored and signal through RET (receptor tyrosine kinase). Thus, absence (as in *PIGA*null hiPSCs) and deficiency (as in *PIGA c*.1234C>T hiPSCs) would be expected to result in a decrease in maintenance and proliferation of neurons. Interestingly, *Gdnf* ^+/-^ mice have severe cognitive deficits similar to humans with inherited GPI anchor deficiency [36]. Alkaline phosphatase, another GPI anchored protein, is important for neuronal development and plays a major role in vitamin B6 metabolism [37]. *Alpl* knockout mice develop intractable seizures that are responsive to administration of vitamin B6. Indeed, Kuki *et al*, described a 9-year-old male with intellectual disability from inherited GPI deficiency due to a germline *PIGO* mutation with vitamin B6 responsive epilepsy [38]. A variety of other GPI anchored proteins are also critical for the developing nervous system. CD56 (NCAM) is important for neurite growth and development [39, 40]. Contactin is an axon-associated adhesion molecule that is important for axon connections in development[41]. Thy-1 [42] and the prion receptor are involved in neurite outgrowth [43]. CD59 is the most important regulator of terminal complement and is widely expressed by the nervous system. Rare patients with congenital, isolated CD59 deficiency have been described [44]. These patients present with a complement-mediated hemolytic anemia and peripheral polyneuropathy. In most cases, the neurologic symptoms are more prominent than the hemolysis, which only becomes clinically relevant during episodes of complement activation, especially during infections. The mechanism behind this observation seems to be the limited neuronal capacity of controlling complement activation because of low neuronal CD59 expression. Administration of eculizumab has been shown to abrogate hemolysis and lead to neuronal regeneration [45]. Neuronal stem cells derived from *PIGA*wt, *PIGA*c.1234C>T, and *PIGA*null hiPSCs showed a dose-response effect in their ability to protect themselves from complement-mediated attack. Complement has been increasingly implicated in a variety of diseases associated with neurodegeneration and intellectual dysfunction [46–48]. Taken together, these data suggest that the neurodegeneration that accompanies inherited GPI anchor deficiency and germline CD59 deficiency may be complement-mediated, and may lend insight into the mechanism of neurodegeneration in macular degeneration [49] and other neurodegenerative diseases [50].

## Conclusion

Here we report on a novel model of inherited GPI anchor deficiency using human iPSCs that provides key insights into the phenotypic features of cognitive disability, neurodevelopmental defects, and neurodegeneration. This model will serve as an important platform in combination with precise gene editing approaches to determine which GPI anchor protein(s) are most responsible for the neuronal defects observed in inherited GPI anchor deficiency.

## Supporting information

S1 Fig Schematic of neuronal differentiation protocol. (TIF)

S2 Fig c.1234C>T mutation decreasing neuron proliferation. Confocal tile scan images (5x5). Four weeks after neuronal differentiation, hNPC-derived neurons from *PIGA*wt and *PIGA*c.1234C>T hNPCs were stained with anti-human GABA (γ-amino butyric acid, green) and anti-human MAP2 (microtubule-associated protein 2, red); nuclei were stained with DAPI (blue). Comparison of hNPC-derived neurons from *PIGA*wt (top) and *PIGA*c.1234C>T (bottom) showed dramatically reduced gro*wt*h in the neurons from the *PIGA*c.1234C>T cell line. Scale bars represent 200 μm.
(TIF)

S3 Fig c.1234C>T mutation causing synapse detect in the neurons from *PIGA*c.1234C>T hNPCs. Confocal fluorescence images of VGAT (green) and Synapsin (red) expression in hNPC-derived neurons from *PIGA*wt and *PIGA*c.1234C>T cell lines. There was a statistically significant decrease density of VGAT and synapse formation in the hNPC-derived neurons from *PIGA*c.1234C>T cells compared to the control cell line (*PIGA*wt). Scale bars represent 20 μm.
(TIF)
