# Supplementary figures and images for: A hypomorphic PIGA gene mutation causes severe defects in neuron development and susceptibility to complement-mediated toxicity in a human iPSC model
Source: PLoS One. 2017 Apr 25;12(4):e0174074. doi: 10.1371/journal.pone.0174074 (PMC5404867; doi:10.1371/journal.pone.0174074)

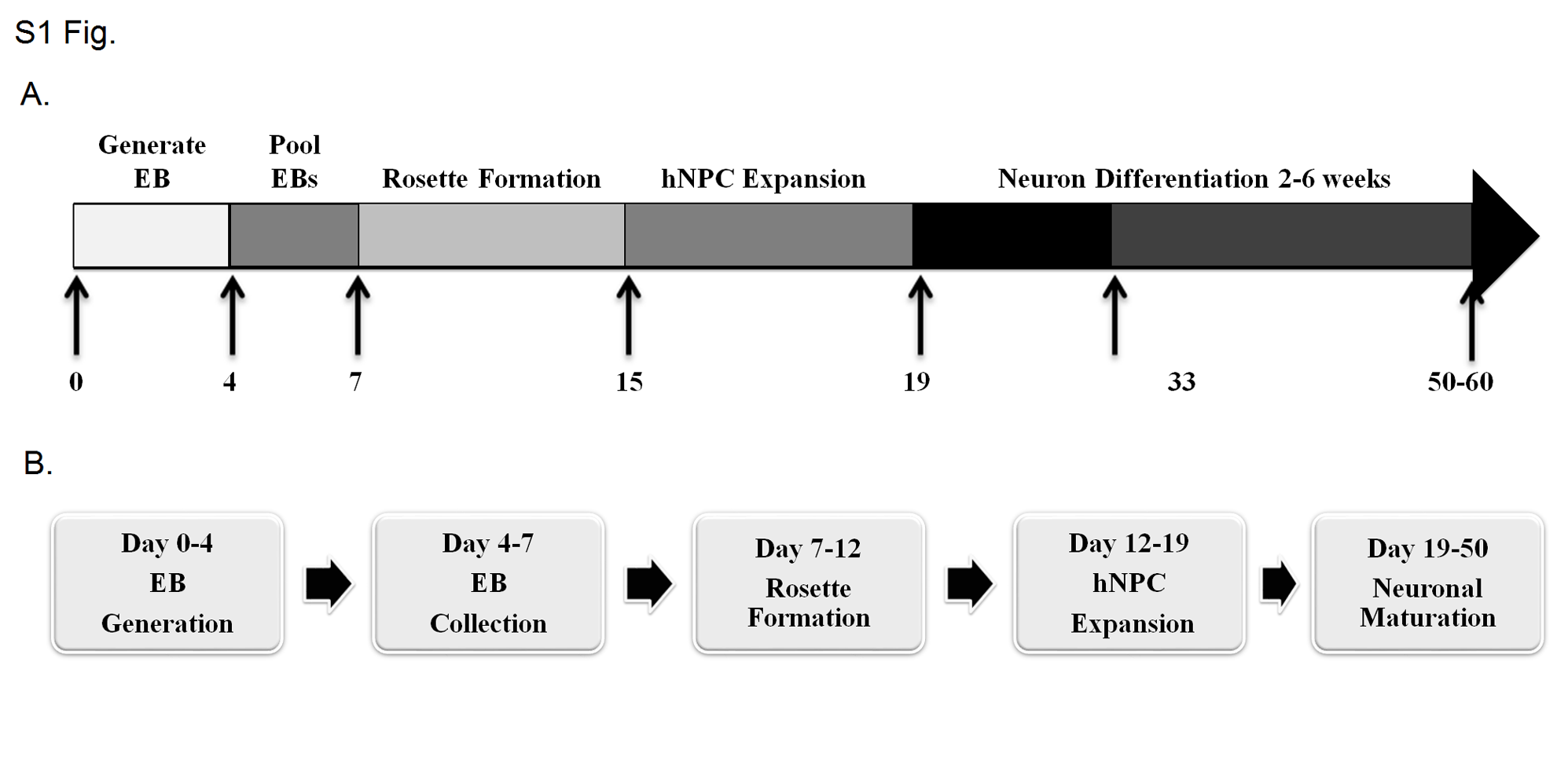

Supplement: S1 Fig — (TIF) [file pone.0174074.s001.tif]

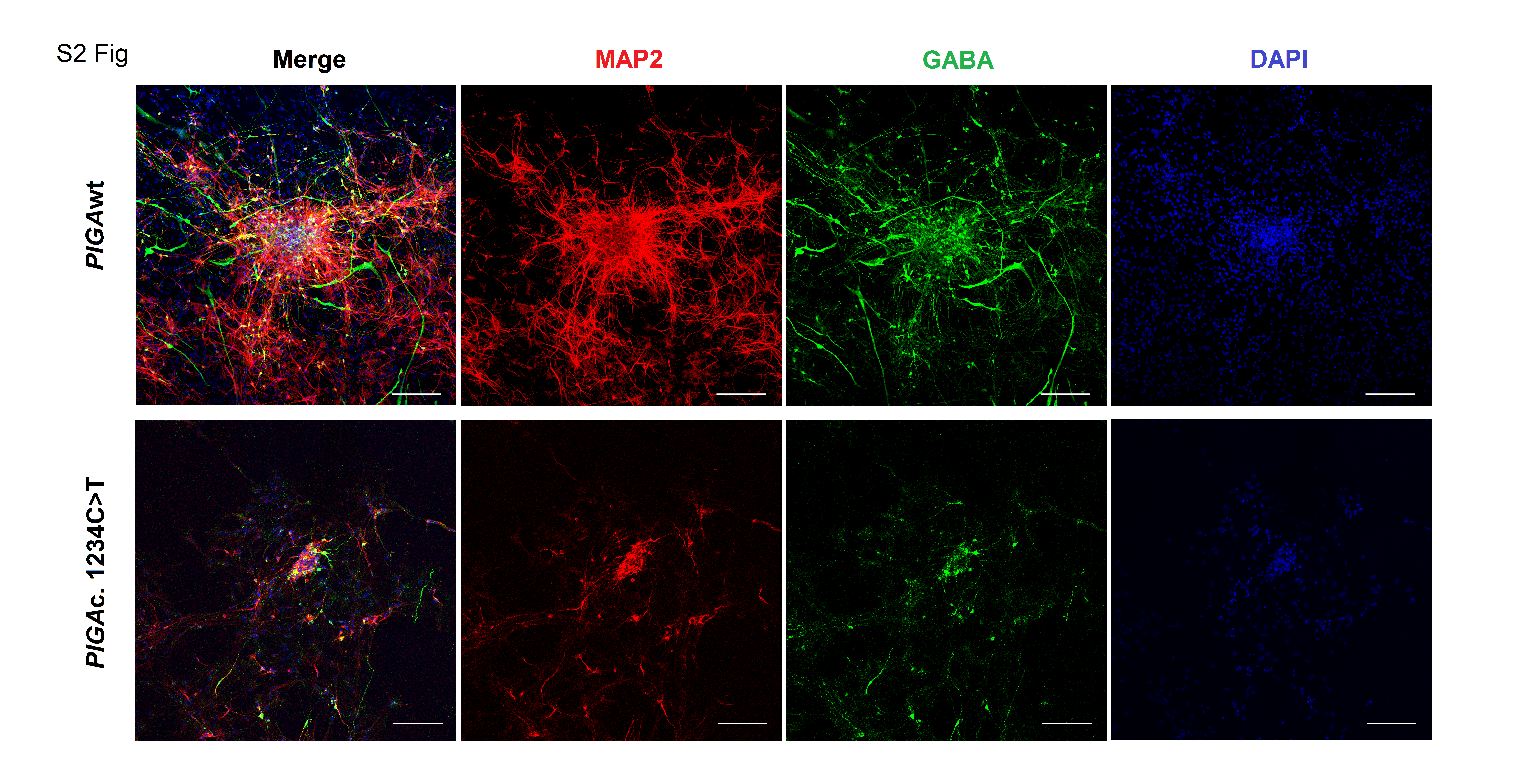

Supplement: S2 Fig — Confocal tile scan images (5x5). Four weeks after neuronal differentiation, hNPC-derived neurons from PIGAwt and PIGAc.1234C>T hNPCs were stained with anti-human GABA (γ-amino butyric acid, green) and anti-human MAP2 (microtubule-associated protein 2, red); nuclei were stained with DAPI (blue). Comparison of hNPC-derived neurons from PIGAwt (top) and PIGAc.1234C>T (bottom) showed dramatically reduced growth in the neurons from the PIGAc.1234C>T cell line. Scale bars represent 200 μm. (TIF) [file pone.0174074.s002.tif]

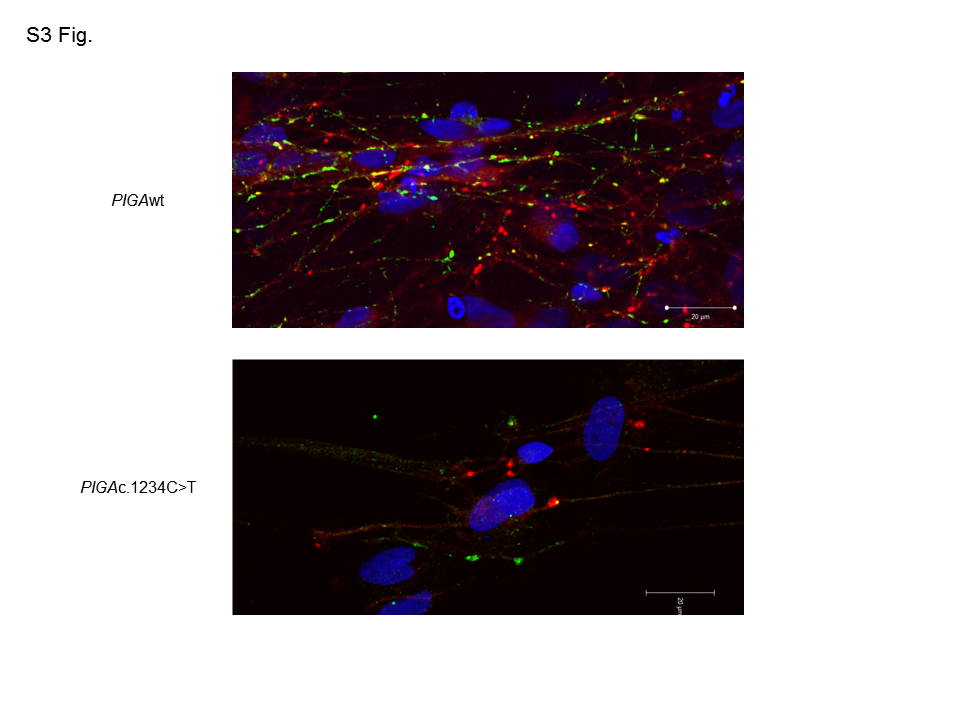

Supplement: S3 Fig — Confocal fluorescence images of VGAT (green) and Synapsin (red) expression in hNPC-derived neurons from PIGAwt and PIGAc.1234C>T cell lines. There was a statistically significant decrease density of VGAT and synapse formation in the hNPC-derived neurons from PIGAc.1234C>T cells compared to the control cell line (PIGAwt). Scale bars represent 20 μm. (TIF) [file pone.0174074.s003.TIF]
